# Preserved learning despite impaired short-term memory in older adults with mild cognitive impairment

**DOI:** 10.3389/fnagi.2025.1560791

**Published:** 2025-03-19

**Authors:** Elaina Smith, Christopher Cortez, April Wiechmann, Sandra Davis, Hannah Dyson, Krystyn Kucharski, Sarah Ross, Geoffrey Kline, Robert T. Mallet, Xiangrong Shi

**Affiliations:** ^1^Department of Pharmacology and Neuroscience, University of North Texas Health Science Center, Fort Worth, TX, United States; ^2^Department of Internal Medicine, University of North Texas Health Science Center, Fort Worth, TX, United States; ^3^Department of Physiology and Anatomy, University of North Texas Health Science Center, Fort Worth, TX, United States

**Keywords:** brief visuospatial memory test, California verbal learning test, digit span test, working memory, learning

## Abstract

**Background:**

The impact of amnestic mild cognitive impairment (aMCI) on short-term memory (STM) and learning performance assessed with different memory modalities was unknown. This study examined differences in STM and learning ability between verbal and visuospatial memory-modalities in older adults with aMCI.

**Methods:**

Thirty-nine aMCI subjects (71.5 ± 6.0 yrs) and 33 non-MCI (control) subjects (71.1 ± 5.7 yrs) of similar age and educational attainment consented to participate in the study. Short-term memory was assessed using Digit-Span-Test (DST), California-Verbal-Learning-Test-2nd edition – short-form (CVLT-II), and Brief-Visuospatial-Memory-Test-Revised (BVMT-R); CVLT-II and BVMT-R assessed verbal-and visuospatial-learning, respectively.

**Results:**

DST-Backward (*p* = 0.016) and DST-Sequencing (*p* < 0.001) scores were significantly lower in the aMCI vs. control subjects (Student’s t-test), but DST-Forward scores did not differ (*p* = 0.237). Immediate and delayed free-recall scores in both CVLT-II (*p* < 0.001) and BVMT-R (*p* < 0.001) were lower in the aMCI subjects. The immediate free-recall scores in both tests improved with repeated trials (two-factor ANOVA: *p* < 0.001 for trial factor) to similar extents in the aMCI and control groups with no significant interaction of the trial and group factors (*p* = 0.266 in CVLT-II and *p* = 0.239 in BVMT-R).

**Significance:**

Amnestic MCI subjects have diminished STM but intact learning ability. Differences in STM of older adults with vs. without aMCI are more readily distinguished by word-verbal memory and/or visuospatial memory testing than digit-verbal memory testing.

## Introduction

Memory is an indispensable cognitive function for carrying out activities of daily living. Memory decline is commonly reported by elderly adults with amnestic mild cognitive impairment (aMCI). In neuropsychologic research and/or geriatric practice, short-term memory (STM), one of the main cognitive domains, is routinely evaluated to assess cognitive function in persons with aMCI. According to Baddeley’s model of working memory (Baddeley, 2012), STM is processed through two sub-systems, the visuospatial sketchpad and the phonological loop, both of which are integrated with and/or commanded by the central executive. Visuospatial memory information is processed by the visuospatial sketchpad sub-system, while verbal memory input, including digit-verbal memory and word-verbal memory, is mediated by the phonological loop sub-system. Short-term memory is often evaluated using verbal-memory tests (Elwood, 1995; Woods et al., 2006; Casaletto et al., 2017), including digit-span (Monaco et al., 2013; Jones and Macken, 2015), and visuospatial memory tests (Benedict et al., 1996; Alescio-Lautier et al., 2007), either separately or in combination.

The possibility that aMCI may affect visuospatial and verbal STM differently remains unresolved. Kessels et al. (2015) reported that digit-span test performance was significantly impaired in MCI subjects and patients with Alzheimer’s disease (AD), while visuospatial memory assessed with spatial span performance did not differ in the MCI vs. control groups. On the other hand, Emrani et al. (2019) found that visual memory assessed with symbol span performance was impaired in MCI vs. non-MCI subjects, while word memory assessed with digit-span backward test did not differ significantly. Furthermore, empirical evidence is lacking regarding the possibility that aMCI may impact digit-word memory and word-verbal memory differently. Assessing cognitive function with different memory modalities in adults with aMCI may afford early, accurate detection of memory decline, and thereby support effective intervention to delay or prevent neurodegenerative progression from aMCI to dementia or AD. Accordingly, one objective of this study was to examine the impact of aMCI on STM in verbal vs. visuospatial memory modalities in older adults.

Recent studies in older adults suggest that cognitive performances in declarative learning (De Wit et al., 2022) is more impaired than procedural learning (De Wit et al., 2021) in aMCI vs. control subjects (De Wit et al., 2023) or in subjects with aMCI or Alzheimer’s disease–dementia vs. healthy controls (Keith et al., 2023). Furthermore, declarative learning performance assessed by California-Verbal-Learning-Test-2nd edition (CVLT-II) varied directly with the volume of medial temporal cortex, whereas procedural learning-retention assessed by modified Trail-Making-Test did not correlate with basal ganglia volume (Keith et al., 2023). Since cognitive assessments with CVLT-II (Kane and Yochim, 2014; Thiruselvam and Hoelzle, 2020; Keith et al., 2023) and Brief-Visuospatial-Memory-Test-Revised (BVMT-R) (Kane and Yochim, 2014; Sawyer et al., 2017; Cai et al., 2023) are commonly applied in neuropsychologic research and/or geriatric practice, and both could evaluate learning and retention/STM simultaneously, the second objective was to compare learning ability in aMCI vs. non-MCI control subjects in different memory modalities. The study hypothesis was that all STM modalities along with learning ability are compromised in aMCI subjects vs. non-MCI control subjects of similar age and educational attainment.

## Materials and methods

### Participants

Thirty-nine subjects (27 women) with aMCI (71.5 ± 6.0 years-old) and 33 subjects (29 women) with normal cognition (71.1 ± 5.7 years-old) gave their written consent and passed a physical screening to participate in the study. The study protocol was reviewed and approved by the North Texas Regional IRB for protection of human subjects. Men and women 55–79 years-old were recruited from the local area through advertisements in senior newsletters and pamphlets placed at regional clinics, or were referred by the Geriatric Center at the UNT Health Science Center. Inclusion criteria included ability to visit the labs for the proposed assessments, depression-free at the time of enrollment, post-menopausal if female, and ≥ 6 months controlled stabilization of chronic conditions including hypertension, coronary artery disease, diabetes or metabolic disease, chronic bronchitis, degenerative osteoporosis/arthritis and/or other aging-related chronic conditions. Exclusion criteria were a diagnosis of AD-dementia, impaired independent daily functioning, mini-mental state exam (MMSE) score < 20 and/or clinical dementia rating (CDR) ≥1; unable to visit the lab independently; active smoker; expecting any major surgery or transplant; having uncontrolled chronic conditions including systolic-diastolic pressures over 150/90 mmHg with medications, diabetes, chronic renal failure, recurrent chest pain, seizures or epilepsies, brain aneurysm, uncontrolled allergic rhinitis, cancer, infectious disease, regular premature ventricular contractions, myocardial ischemia or infarct, severe head injury or traumatic brain injury, stroke, currently diagnosed depression, or having metallic implants above the neck or active in nature (e.g., cardiac pacemaker, stimulators, infusion pumps).

**Figure 1:**
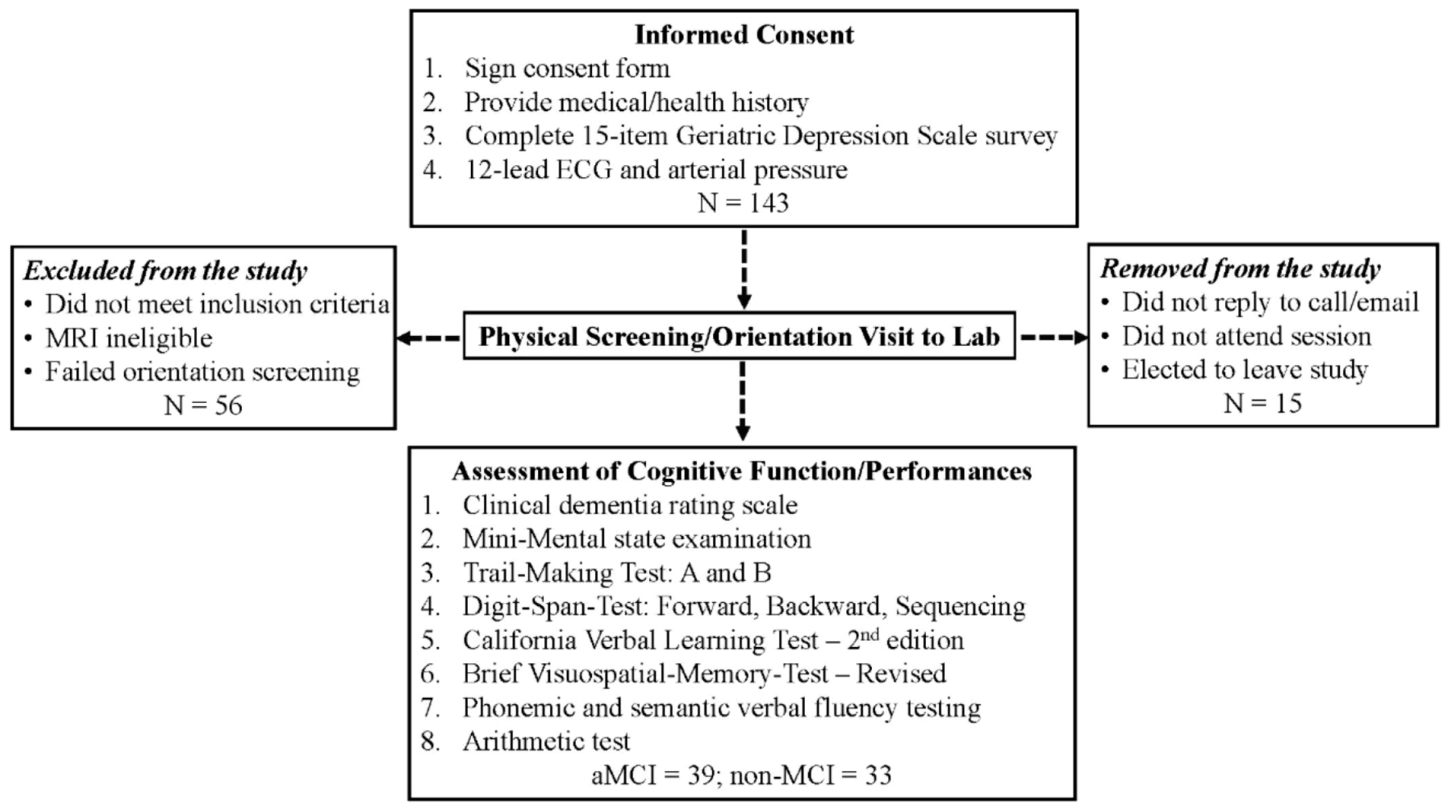
Flowchart for subject enrollment and testing procedure.

Each subject’s cognitive function including CDR was assessed by a geriatric psychiatrist or neuropsychologist at the Geriatric Center of the UNT Health Science Center. Subjects having a self-or family member-reported memory complaint, whose CDR was ≤0.5, and/or whose testing scores in one or more STM modalities were ≥ 1 standard deviation below the age-and education-adjusted group averages, were determined to have aMCI based on the criteria described previously (Winblad et al., 2004; Petersen et al., 2010). Figure 1 summarizes subject enrollment and testing procedures. All subjects spoke English, were free of clinical depression, psychiatric disorders and neurologic dysfunction based on the medical history survey and sustained normal daily functionality. Most of these subjects had some college education (Table 1).

**Table 1:** Group basic characteristics.

	MCI (*n* = 39)	Normal (*n* = 33)	*t* value	*p*	Cohen’s D
Men vs. Women	12:27	4:29	–	0.087^*^	–
Age (year)	71.5 ± 6.0	71.1 ± 5.7	0.27	0.790	0.15
Education (year)	16.0 ± 1.9	16.2 ± 2.1	−0.47	0.643	0.15
CDR (point)	0.5 ± 0.0	0.3 ± 0.2	3.26	<0.001	0.35
MMSE (point)	27.6 ± 1.5	28.7 ± 1.1	−3.27	0.002	0.89
GDS (point)	1.2 ± 1.3	1.1 ± 1.3	0.57	0.571	0.15
Weight (kg)	72.2 ± 15.9	74.9 ± 13.9	−0.78	0.440	0.71
Height (cm)	166 ± 10	166 ± 9	0.12	0.907	0.08
SBP (mmHg)	136 ± 9	134 ± 12	0.92	0.361	0.70
DBP (mmHg)	75 ± 11	77 ± 9	−1.07	0.287	0.81
MAP (mmHg)	95 ± 9	96 ± 9	−0.45	0.651	0.32
HR (bpm)	67 ± 12	67 ± 10	0.11	0.911	0.09

Number (%) of subjects with medication					
Category of medication	aMCI	Normal	Total	*p*	–
Hypertension/Coronary arterial disease	20 (27.8)	12 (16.7)	32 (44.4)	0.240	–
Hyper-cholesterol/Hyperlipidemia	18 (25.0)	11 (15.3)	29 (40.3)	0.338	–
Hyperglycemia	9 (12.5)	3 (4.2)	12 (16.7)	0.203	–
Anxiety/Depression	15 (20.8)	9 (12.5)	24 (33.3)	0.452	–
Reflux/Gastric acid	10 (13.9)	10 (13.9)	20 (27.8)	0.793	–
Hypothyroid/Hyperthyroid	8 (11.1)	12 (16.7)	20 (27.8)	0.188	–
Allergy	10 (13.9)	8 (11.1)	18 (25.0)	1.000	–
Sleep aid	7 (9.7)	4 (5.6)	11 (15.3)	0.533	–
Hormone replacement	6 (8.3)	4 (5.6)	10 (13.9)	0.745	–

CDR, clinical dementia rating; MMSE, mini-mental state examination; GDS, geriatric depression scale (15-point short form); SBP, systolic blood pressure; DBP, diastolic blood pressure; MAP, mean arterial pressure; HR, heart rate. Values are group means ± standard deviation of the mean (SD). ^*^denotes the test of men vs. women ratio between two groups using Fisher’s exact test for two-tailed probability. All other outcomes based on the TTEST procedure for two independent groups. Cohen’s D presents the absolute value.

### Cognition assessment

Cognitive performance was assessed in-person in a quiet testing room. Mini-Mental State Examination (MMSE PAR^®^ Lutz, FL) was performed for global cognitive assessment. Times to completion of the Trail-Making Test versions A and B (TMT-A and TMT-B) were recorded to evaluate attention and executive function. Phonemic verbal fluency and semantic verbal fluency were assessed with the words beginning with letters F-A-S and the animal names said by subjects within 60 s, respectively. Arithmetic test was administrated using 22 items from Wechsler Adult Intelligence Scale® – 4th edition (WAIS®-IV PEARSON, Bloomington, MN). Correct recall numbers of Digit-Span-Test (DST) Forward, Backward and Sequencing (WAIS®-IV PEARSON, Bloomington, MN) were documented to assess digit-verbal memory. Each sub-test began with three numbers and each subject undertook two attempts per span, which increased by 1 digit with 8 increments and ended after the subject failed both attempts with the same digits. DST-Forward and DST-Backward requested the subject to state the numbers in the same order forward and backward, respectively. DST-Sequencing asked the subject to state the numbers in ascending order. Maximal score was 16 points for each DST subtest (i.e., 2 trials/span x 8 spans). Recall of the terms in four trials of immediate free-recall (FR), short-delayed (30-s) FR and long-delayed (10-min) FR of CVLT-II Short-Form (PEARSON, Bloomington, MN) were documented to assess learning (more likely declarative or non-procedural learning) and word-verbal memory. A perfect score was nine words in all three sub-categories. Three BVMT-R (PAR® Lutz, FL) trials of immediate-recall and 30-min delayed-recall were conducted to evaluate learning (more likely procedural or non-declarative learning) and visuospatial memory, with a maximal score of 12 points (6 line-sketches x 2 points/sketch). These STM modality tests are well accepted in the field and were applied in our pilot study (Wang et al., 2020). All the tests were completed in one ≤75 min session. Only raw scores were reported and compared between the aMCI and control groups.

### Data analysis

Scalar values from the aMCI and control groups were compared by Student’s t-tests. Cohen’s D between-group effect size (absolute value) for t-test equaled (M1 – M2)/√[(SD1 + SD2)/2], where M = mean value and SD = standard deviation. Two-factor ANOVA was applied to test the significance of the group factor (i.e., aMCI vs. control) and trial factor (i.e., trials 1–4 in CVLT-II and trials 1–3 in BVMT-R) along with the interactions of the group and trial factors (for assessing learning effect) using the general linear model procedure. Furthermore, verbal-learning and visuospatial-learning were also estimated from the differences between trial 1 raw score and the higher of the raw scores from trials 3 and 4 in CVLT-II free-recalls and the raw scores from trials 2 and 3 in BVMT-R free-recalls, respectively. Data are reported as group mean values ± SD of the mean. *p* values ≤0.05 were taken to indicate statistical significance. Statistical Analysis System® software (Cary, NC) was used for the data analyses.

## Results

**Figure 2:**
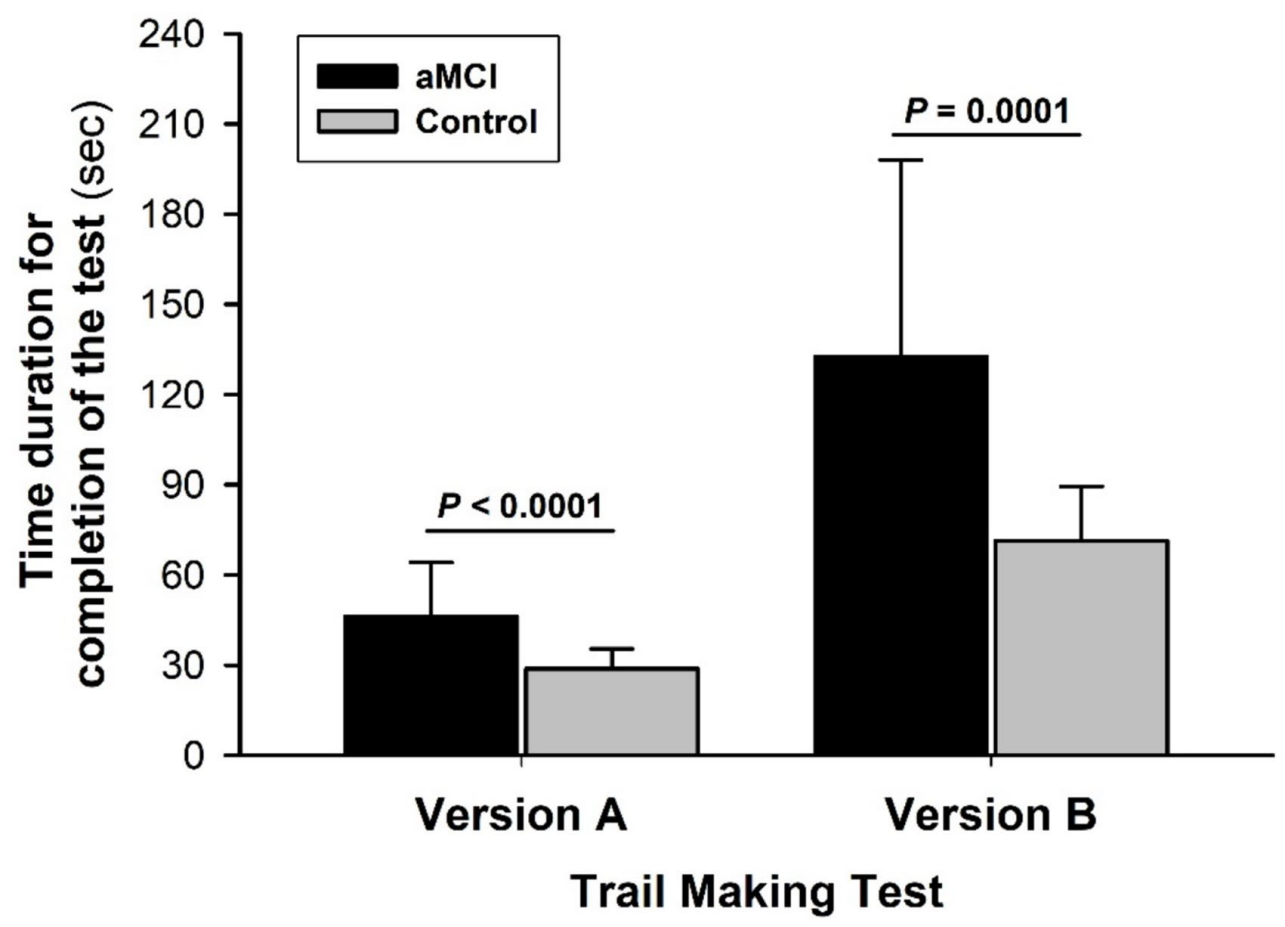
Trail making test of attention and executive function. The times required to complete TMT-A and TMT-B were significantly longer in the MCI than non-MCI (control) groups. Both group factor (*F* = 36.13, *p* < 0.0001) and subtest factor (*F* = 109.74, *p* < 0.0001) significantly affected performance time with a significant interaction of the group and subtest factors (*F* = 12.19, *p* < 0.001). A greater disparity between aMCI and control subjects emerged from TMT-B vs. the less challenging TMT-A, suggesting reduced mental flexibility or adaptability in the aMCI group. Values in this and the other figures are mean ± SD from 39 aMCI and 33 control subjects.

There were no significant differences in group age or education attainment between the aMCI and control subjects (Table 1). Both groups had a similar health and medication history. MMSE scores were slightly but significantly lower in the aMCI vs. non-MCI groups. Times to test completion were prolonged in the aMCI vs. control subjects in both TMT-A: 46.2 ± 17.9 vs. 28.8 ± 6.6 s (*t* = 5.59, *p* < 0.001, Cohen’s D = 4.97) and TMT-B: 132.6 ± 65.5 vs. 71.4 ± 18.1 s (*t* = 5.46, *p* < 0.001, Cohen’s D = 10.23), indicating impaired performance (Figure 2). The difference in completion time between TMT-B and TMT-A was appreciably greater (*t* = 4.28, *p* < 0.001, Cohen’s D = 7.19) in the MCI (+87.4 ± 61.2 s) vs. control (+42.5 ± 16.6 s) subjects with a significant interaction of the group and subtest factors (*F* = 12.19, *p* < 0.001). Overall, aMCI subjects had lower scores in both verbal fluency and arithmetic tests (Table 2).

**Table 2:** Performance of verbal fluency and arithmetic test.

Testing	MCI (*n* = 15)	Normal (*n* = 15)	*t* value	*p*	Cohen’s D
Phonemic verbal fluency	34 ± 12	43 ± 14	−1.93	0.063	2.56
Semantic verbal fluency	17 ± 7	23 ± 10	−1.93	0.064	2.04
Arithmetic test	8 ± 2	10 ± 2	−2.61	0.015	1.36

Values are group mean ± SD. Cohen’s D presents the absolute value.

### Digit-verbal memory

**Figure 3:**
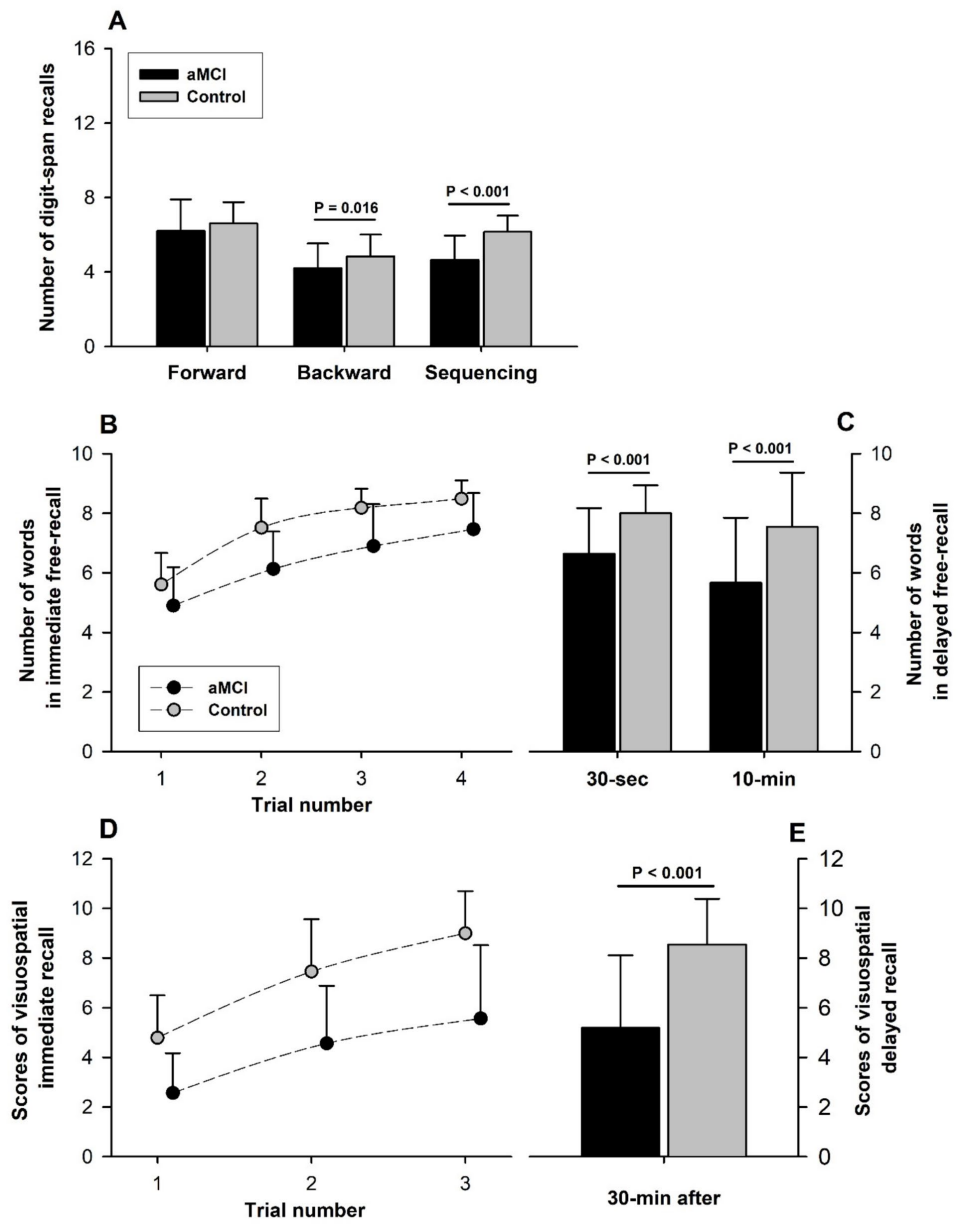
Assessment of verbal and visuospatial memory. **(A)** Although DST-Forward test scores were not significantly different between the aMCI and control groups, DST-Backward and DST-Sequencing scores were significantly lower in the aMCI vs. control group. Overall, scores were lower in DST-Backward than DST-Forward (*F* = 64.58, *p* < 0.001 for subtest factor) with a significant group factor (*F* = 6.19, *p* = 0.014), but no significant interaction of the group and subtest factors between DST-Forward and DST-Backward (*F* = 0.53, *p* = 0.466). A raw score of 16 is the perfect score for each of the three subtests. **(B)** All scores across different trials between the groups were significantly different (Group factor: *F* = 70.01, *p* < 0.001). Scores of immediate free-recall (FR) tests in CVLT-II (Perfect score: 9 words/trial) improved steadily with successive trials in both the aMCI and control subjects (Trial factor: *F* = 82.50, *p* < 0.001). Score improvement over multiple trials was similar in the aMCI and control subjects (*F* = 1.33, *p* = 0.266 for interaction of the group and trial factors), suggesting similar verbal-learning in the two groups. **(C)** Short-and long-delayed FR scores were significantly lower in the aMCI than non-MCI subjects, indicating verbal memory impairment associated with aMCI. 10-min FR score was significantly lower than 30-s FR score only in the MCI group. **(D)** All visuospatial testing scores of immediate recalls (12 points/trial for a perfect score) were significantly lower in the aMCI than control groups (*F* = 95.24, *p* < 0.001 for group factor), indicating impaired visuospatial memory associated with aMCI. The scores of Immediate recall scores improved with each successive trial in both groups (Trial factor: *F* = 52.29, *p* < 0.001). The extent of score improvement over successive trials was not significantly different between the groups (Interaction of group and trial factors: *F* = 1.44, *p* = 0.239), suggesting similar visuospatial-learning in the aMCI and normal subjects. **(E)** 30-min delayed recall was lower (*t* = −5.92, *p* < 0.001) in the aMCI (5.2 ± 2.9) than the control subjects (8.5 ± 1.8).

DST-Forward scores were not different (*t* = −1.19, *p* = 0.237, Cohen’s D = 0.34) between the aMCI (6.2 ± 1.7) and control (6.6 ± 1.1) subjects; DST-Backward and DST-Sequencing scores were significantly lower in the aMCI vs. control subjects: 4.2 ± 1.3 vs. 4.9 ± 1.2 (*t* = −2.47, *p* = 0.016, Cohen’s D = 0.66) for DST-Backward and 4.6 ± 1.3 vs. 6.2 ± 0.9 (*t* = −5.84, *p* < 0.001, Cohen’s D = 1.45) for DST-Sequencing (Figure 3A). Overall, the DST-Backward scores were significantly lower than DST-Forward in both groups (*F* = 64.58, *p* < 0.001 for subtest factor) and the group factor was significant (*F* = 6.19, *p* = 0.014), but no interaction of the group and subtest factors was detected (*F* = 0.53, *p* = 0.466).

### Word-verbal memory

CVLT-II immediate free-recall (FR) scores were consistently superior in the control vs. MCI subjects (*F* = 70.01, *p* < 0.001 for group factor) with all between-group differences from FR-1 to FR-4 being statistically significant. These scores for word-verbal memory performance improved significantly with trial repetition (*F* = 82.50, *p* < 0.001 for trial factor) in both groups, indicating a significant learning effect (Figure 3B). The rate of performance improvement with repeated CVLT-II immediate FR trials was similar in the groups (*F* = 1.33, *p* = 0.266 for interaction of the trial and group factors), indicating similar verbal-learning in the aMCI and control subjects. Furthermore, learning scores of CVLT-II (the higher of the trial 3 and 4 scores minus the trial 1 score) were not different (*t* = −1.04, *p* = 0.300, Cohen’s D = 0.27) between the aMCI (2.7 ± 1.3) and control (3.0 ± 1.0) subjects. Both 30-s short-delayed FR and 10-min long-delayed FR scores were significantly lower in the aMCI vs. control subjects (short-delayed FR: aMCI 6.6 ± 1.5 vs. control 8.0 ± 0.9 [*t* = −4.62, *p* < 0.001, Cohen’s D = 1.22]; long-delayed FR: aMCI 5.7 ± 2.2 vs. control 7.5 ± 1.8 [*t* = −3.92, *p* < 0.001, Cohen’s D = 1.33]), indicating aMCI-associated impairment of verbal STM (Figure 3C). Furthermore, the long-delayed FR score fell significantly below the short-delayed score in the aMCI (−1.0 ± 1.7; *t* = −3.64, *p* < 0.001) but not the control subjects (−0.5 ± 1.5; *t* = −1.76, *p* = 0.087).

### Visuospatial memory

The BVMT-R scores of immediate-recall were consistently lower in the aMCI vs. control subjects (*F* = 95.24, *p* < 0.001 for group factor), but improved significantly with successive trials (*F* = 52.29, *p* < 0.001 for trial factor) in both groups (Figure 3D). The interaction of the group and trial factors was not statistically significant (*F* = 1.44, *p* = 0.239), indicating no differences in visuospatial-learning in MCI vs. control subjects. But the BVMT-R learning score (the higher of the trial 2 and 3 scores minus the trial 1 score) was lower (*t* = −2.41, *p* = 0.019, Cohen’s D = 0.77) in the aMCI (3.2 ± 2.1) vs. control (4.3 ± 1.5) groups. The 30-min delayed-recall score was significantly lower (*t* = −5.92, *p* < 0.001, Cohen’s D = 2.18) in the aMCI (5.2 ± 2.9) than control (8.5 ± 1.8) subjects (Figure 3D), indicating a reduced visuospatial STM in the MCI subjects.

## Discussion

This study is the first to define the impact of aMCI on verbal and visuospatial STM in different memory modalities. Although digital-verbal memory in DST-Forward was not significantly different between the groups, testing word-verbal memory with CVLT-II or visuospatial memory with BVMT-R distinguished STM impairment in older adults with aMCI vs. age-matched non-MCI controls. Furthermore, our data suggested that cognitive learning, especially verbal-learning (declarative or non-procedural learning), seemed to be preserved in the aMCI subjects despite their diminished STM. Both TMT-A and TMT-B revealed decreased attention, executive function, and/or coordinated visual-motor processing speed in the aMCI group. Overall, aMCI was associated with diminished performance in MMSE, verbal fluency and arithmetic tests.

Visuospatial memory information is transferred by the visual cache and the inner scribe through the visuospatial sketchpad sub-system to the central executive, which coordinates with the visual-motor efferent output. Input of DST and CVLT-II is processed by the auditory-verbal afferent pathway through the phonological loop sub-system to the central executive that provides the command for the articulatory or phonological output. The present results suggest that aMCI may negatively impact both the visuospatial sketchpad and phonological loop sub-systems. However, not all DST subtests showed significant differences in digit-verbal memory between the aMCI and control subjects. On the other hand, the word-verbal memory testing scores from immediate recall trials 1 to 4, and 30-s and 10-min interval delayed recalls were all significantly lower in the aMCI subjects vs. cognitively normal controls. The digit-verbal and word-verbal memory modalities are proposed to be processed by the same phonological loop sub-system (Baddeley, 2012). The reasons for the lower aMCI sensitivity of digit-verbal memory evaluated by DST-Forward vs. CVLT-II-assessed word-verbal memory are not well understood but may be related to the less semantic character of numerical digit-span vs. word recalls.

When comparing the performances in the two DST subtests, both the aMCI and control subjects had lower scores in DST-Backward than DST-Forward (Figure 3). These results were concordant with a previous report that both of these DST scores declined with aging, but the DST-Backward scores were consistently below the DST-Forward scores in adult subjects aged 20 to 90 years (Monaco et al., 2013). The differences between these DST subtests were proposed to have resulted from greater taxation of the central executive (Monaco et al., 2013) and/or greater working memory demands (Coalson et al., 2010) imposed by the DST-Backward procedure. However, neither age in the previous study (Monaco et al., 2013) nor aMCI in the current study (Figure 3) affected the difference in DST-Forward between the groups, suggesting that testing with DST-Forward may be not sensitive to distinguish a difference in verbal-STM.

On the other hand, the central executive function and cortical-visual-motor coordinated processing speed were significantly diminished in the aMCI subjects, as evidenced by their poorer performance in both TMT-A and TMT-B. In this study, time to completion of TMT-B exceeded that of TMT-A in both MCI and control subjects. However, the prolongation of TMT-B vs. TMT-A was significantly greater in the aMCI than control subjects, suggesting that mental flexibility declined in the aMCI subjects along with their attention and central executive functions. Furthermore, both verbal fluency and arithmetic tests along with MMSE scores tended to be lower in the aMCI subjects compared to the control subjects, suggesting that aMCI shows a trend to impair global cognition and/or long-term retention-retrieve function (Figure 3).

Although central executive function and STM were diminished with aMCI, the improvements in CVLT-II (Figure 3B) and BVMT-R (Figure 3D) with repeated trials did not differ significantly in the aMCI vs. control subjects, suggesting comparable cognitive learning ability in the two groups, both CVLT-II (declarative or non-procedural) learning and BVMT-R (procedural or non-declarative) learning modalities. Thus, learning ability seemed to be preserved despite decreased STM in the older adults with the transient pre-dementia state of aMCI. However, testing applied in this study focused on assessing STM, and *learning ability* was only estimated from the differences between repeated CVLT-II and BVMT-R. Therefore, our data did not indicate any impairment in long-term memory (LTM) or the ability to consolidate STM into LTM/retention-retrieve in the subjects with aMCI vs. normal cognition. Other study limitations include small sample size in this cross-sectional study, and the absence of neuroanatomic and neurophysiological correlates of cognitive function. Longitudinal studies with large sample sizes are needed to correlate changes in learning ability and STM with neurologic mechanisms of aMCI, and to determine if these early findings are predictors of progression to AD-dementia.

Our data indicate that visuospatial memory and word-verbal memory testing seem more sensitive than digit-verbal memory testing to detect STM differences in subjects with aMCI vs. control cognition, with implications for assessing aMCI-related STM decline in geriatric practice and/or neurocognitive research. Although the aMCI subjects showed decreased STM, executive function with processing speed, and global cognition, their learning ability, especially verbal learning (declarative or non-procedural learning) seemed to be unimpaired vs. their non-MCI counterparts. Because the preserved learning indicates that aMCI adults may remain trainable, memory training intervention at this early stage of cognitive decline may help delay or prevent STM deterioration, or slow its progression. In conclusion, although verbal and visuospatial memory functions are significantly diminished in older adults with MCI, *learning ability* may be preserved. Verbal memory evaluation with DST-Forward may not be sufficiently sensitive to detect aMCI-related differences in STM.

## Data Availability

The original contributions presented in the study are included in the article/supplementary material, further inquiries can be directed to the corresponding author.
